# The effect of oil pulling with coconut oil to improve dental hygiene and oral health: A systematic review

**DOI:** 10.1016/j.heliyon.2020.e04789

**Published:** 2020-08-27

**Authors:** Julian Woolley, Tatjana Gibbons, Kajal Patel, Roberto Sacco

**Affiliations:** a King's College Hospital, King's College NHS Foundation Trust, UK; b John Radcliffe Hospital, Oxford University Hospital Trust, UK; c University of Manchester Division of Dentistry Manchester, UK

**Keywords:** Dental surgery, Dentistry, Periodontics, Oral medicine, Alternative medicine, Coconut oil, Oil pulling, Ayurvedic medicine, Oral health, Dental hygiene

## Abstract

**Objectives:**

Coconut oil is a cheap and accessible oil for many people around the world. There are numerous advocates for the practice of oil pulling to prevent common oral diseases. Therefore determining the effectiveness of oil pulling with coconut oil could potentially have monumental benefits. This review aimed to assess the effect of oil pulling with coconut oil in improving oral health and dental hygiene.

**Data:**

We included randomized controlled trials comparing the effect of oil pulling with coconut oil on improving oral health and dental hygiene.

No meta-analysis was performed due to the clinical heterogeneity and differences in the reporting of data among the included studies.

**Sources:**

Six electronic databases were screened: PubMed, Medline, EMBASE, AMED, CENTRAL and CINAHL.

**Study selection:**

Electronic searches yielded 42 eligible studies, of which four RCTs including 182 participants were included. The studies lasted between 7 and 14 days. Significant differences were demonstrated for a reduction in salivary bacterial colony count (*p* = 0.03) and plaque index score (*p*=<0.001). One study also demonstrated a significant difference in staining compared to using Chlorhexidine (*p* = 0.0002). However, data was insufficient for conclusive findings, the quality of studies was mixed and risk of bias was high.

**Conclusion:**

The limited evidence suggests that oil pulling with coconut oil may have a beneficial effect on improving oral health and dental hygiene. Future clinical trials are of merit considering the universal availability of the intervention. Prospective research should have a robust design with rigorous execution to provide a higher quality of evidence.

**Clinical significance:**

Oil pulling with coconut oil could be used as a adjunct to normal preventative regimes to improve oral health and dental hygiene although further studies are needed to determine the level of effectiveness.

## 1 Introduction

Oral hygiene habits are developed and established in early childhood and aid in the prevention of dental caries and periodontal disease in the future. Mechanical methods of tooth brushing are the most reliable and widely accepted, however mouthwashes have also been used for a number of years as an adjunctive measure for the maintenance of dental hygiene and oral health [1].

Oil pulling is a traditional ayurvedic remedy originally practised in ancient India for the maintenance of oral health. It is thought to cure over thirty systemic diseases as well as conferring multiple oral health benefits such as improvement in gingival health with reduced inflammation and bleeding, resolution of symptoms of dry mouth/throat and chapped lips, whiter teeth, reduced halitosis, improved oral hygiene and strengthening of muscles and jaws in the oral cavity [2]. The procedure of oil pulling involves swishing a measured volume of oil around the mouth for a period of time, forcing the oil in between all the teeth and around the mouth [2]. Examples of organic oils that are used include sunflower oil, sesame oil, and coconut oil [2].

Coconut oil is composed mostly of medium chain fatty acids; it is therefore unique compared to the majority of other dietary oils, which are predominantly made up of long chain fatty acids. Approximately 50% of these medium-chain fatty acids are lauric acid, known for its antimicrobial and anti-inflammatory benefits [3]. Previous in-vitro studies using biofilm models have demonstrated the antimicrobial properties of coconut oil against Streptococcus mutans and Candida albicans [4].

As coconut oil is a readily accessible and cheap material for most, research into the effectiveness and efficacy of its use in the oil pulling procedure is of clinical merit. As there have been no previous systematic reviews undertaken specifically for coconut oil use in oil pulling, the aim of this systematic review is to assess the available evidence and effectiveness of this ayurvedic remedy in improving the oral health and dental hygiene. This review has potential to offer another dimension in the role of alternative medicine within dentistry.

## 2 Materials and methods

This systematic review was conducted according to Preferred Reporting Items for Systematic Reviews and Meta-Analyses (PRISMA) guidelines [5]. The protocol of this review was registered in the International Platform of Registered Systematic Review and Meta-analysis Protocols (INPLASY) under number INPLASY202060084.

### 2.1 Review question and PICO strategy

Is there sufficient evidence that coconut oil when used in an oil pulling technique improves oral health and dental hygiene?

⁃ Population (P): any human participant
⁃ Intervention (I): oil pulling with coconut oil
⁃ Comparison (C): conventional oral hygiene routines and alternative evidence-based interventions
⁃ Outcome (O): effect on oral health and dental hygiene

### 2.2 Information sources and search strategy

**Figure 1:**
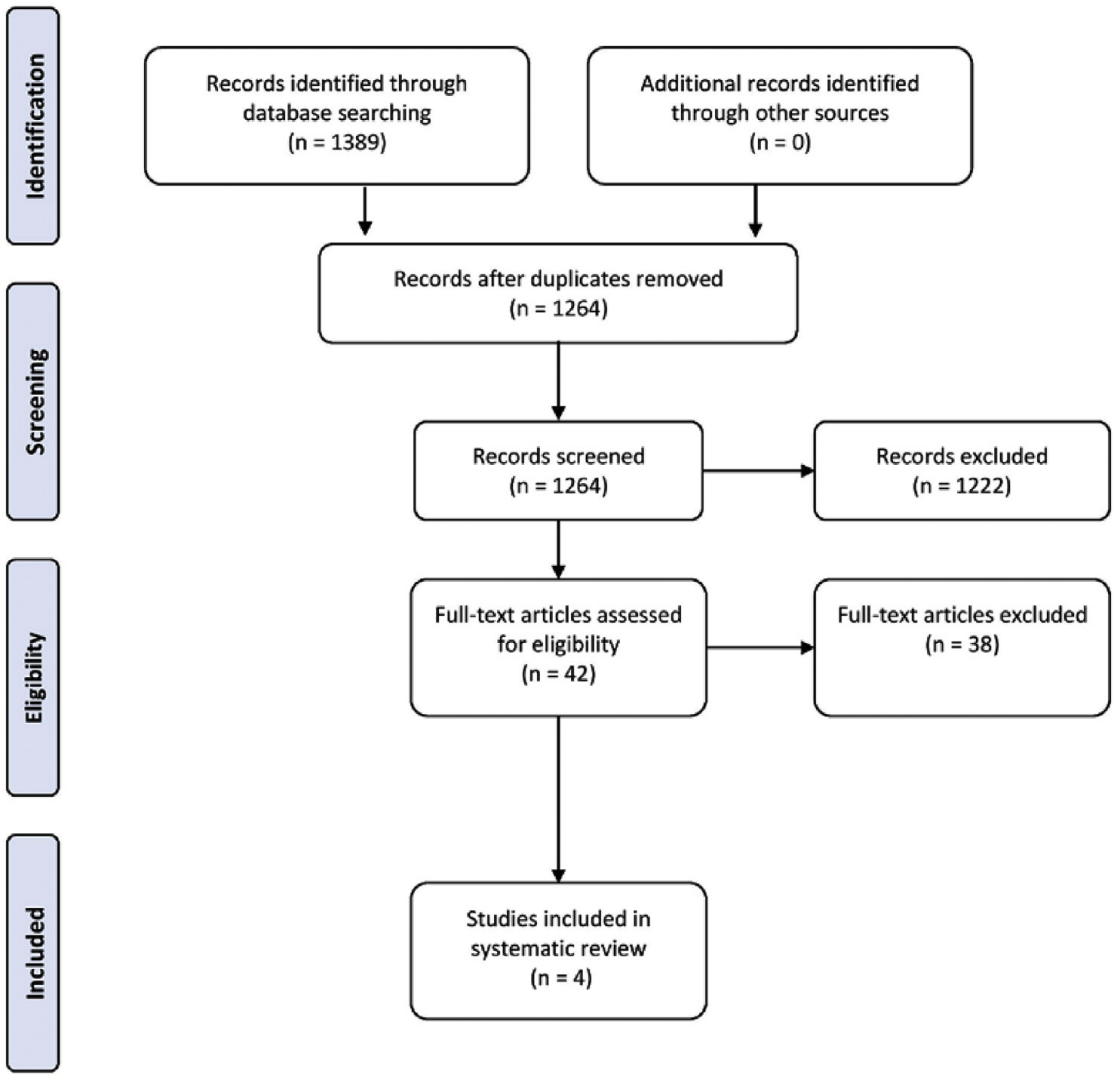
Search strategy used to collect articles for systematic review.

The following six databases were screened: PubMed, Medline, EMBASE, AMED, CENTRAL and CINAHL (Figure 1). A comprehensive search strategy for all six databases was developed focussing on Ayurvedic medicine in conjunction with oral health: Periodontal OR Periodont OR Periodontitis OR Gingivitis OR Gingival OR Periodontal disease OR Periodontics OR Oral OR Dental OR Oral health OR Oral hygiene OR Dental hygiene OR Halitosis AND Coconut pulling OR Coconut oil OR Oil pulling OR Ayurveda OR Ayurvedic medicine. The search strategy included appropriate changes in the keywords and followed syntax rules for each of the six databases.

A comprehensive screening method was employed to ensure precision within the search. One of the authors (JW) identified and removed duplicates. The screening of titles and abstracts were carried out independently by two authors (JW and TG) to eliminate any irrelevant material. Disagreements were resolved by discussion until a consensus was reached. If conflicts were not resolved, the studies were sent forward to a third reviewer for resolution (KP). Finally two authors (JW and TG) conducted full-text screening and completed data extraction using a predefined and standardised Microsoft Excel form to:

- Verify the study eligibility derived from the inclusion/exclusion criteria.
- Extract data on study characteristics and outcomes for the included studies.
- Carry out a methodological quality assessment and risk-of bias assessment.

The authors of any studies eligible for inclusion in the review with insufficient information were contacted directly using e-mail. Where pooling of analogous data was inappropriate, the results of the trials were reported as a narrative description using detailed commentary on the study findings, interventions and controls and outcomes. No meta-analysis was performed due to the clinical heterogeneity and differences in the reporting of data among the included studies.

### 2.3 Criteria for inclusion

Studies included in the research strategy, included published or unpublished randomised controlled trials. The last updated search was performed in June 2020. No restrictions were imposed regarding year/time of publication to maximise the pool of appropriate studies. No restriction of age, gender, sample size or ethnic origin was applied. There were no language restrictions enforced on the search.

Animal studies, in vitro studies, studies without a randomised-controlled design, reviews and studies not using coconut oil as an intervention were excluded.

### 2.4 Objectives

The objective of this review was to appraise all data from randomised controlled trials to determine whether there is sufficient evidence that coconut oil when used in an oil pulling technique improves dental hygiene and oral health compared to other conventional and evidence-based interventions.

### 2.5 Outcomes measured

The primary outcome was to determine whether oil pulling with coconut oil improves oral health.

The secondary outcomes were to determine whether the duration of use and method of delivery of coconut oil affect oral health and dental hygiene. In addition, the review sought to compare this to alternative conventional interventions.

### 2.6 Data extracted

All selected papers were carefully read to identify author(s), year of publication, study design, population sample, interventions and oral hygiene adjustments. To assess our primary outcome, all data corresponding to oral health measures were extracted from the studies including plaque index (PI), gingival index (GI), stain index (SI), bleeding on probing (BOP), salivary *Streptococcus Mutans* (*SM*) count and salivary bacterial colony (BC) count.

### 2.7 Risk of bias and review of quality assessment

**Figure 2:**
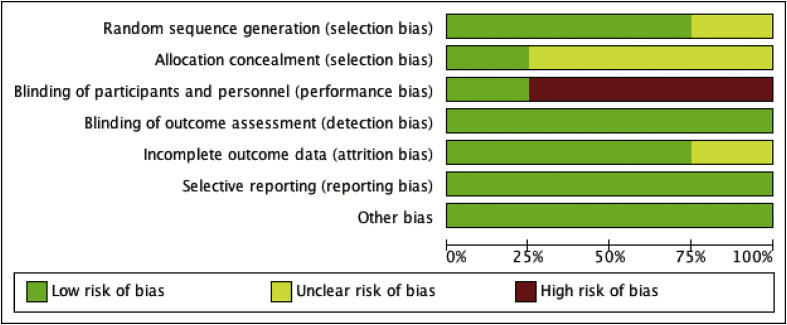
Risk of bias graph: review authors' judgements about each risk of bias item presented as percentages across all included studies.

**Figure 3:**
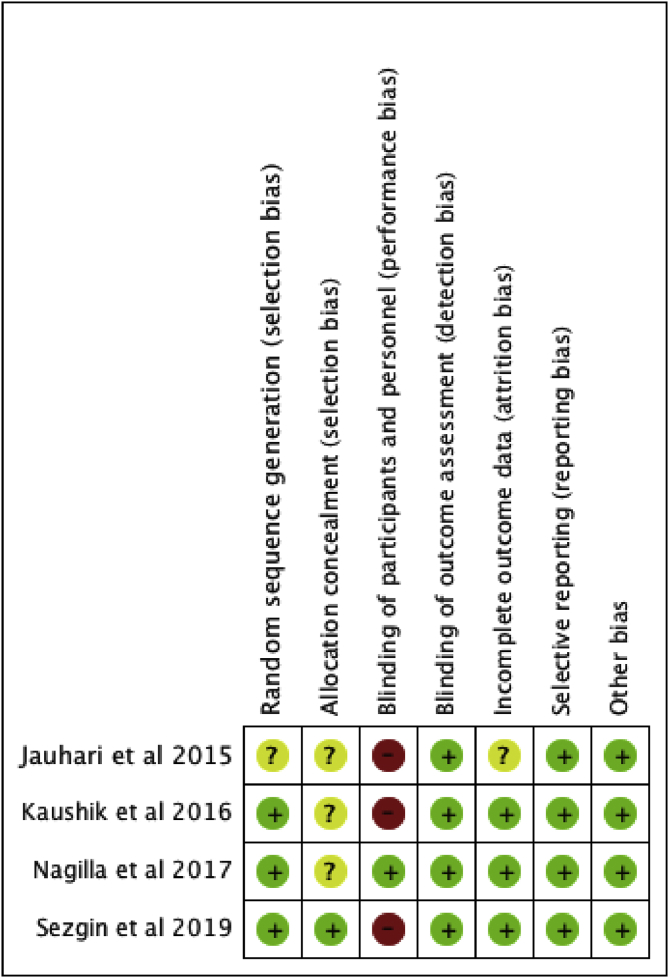
Risk of bias summary: review authors' judgements about each risk of bias item for each included study.

Two authors (JW and TG) independently appraised the risk of bias in this review. The Cochrane Handbook for Systematic Reviews of Interventions was used to appraise the risk for each randomised controlled trial (Figures 2 and 3) [6]. In addition, the quality of included studies was assessed according to the levels of evidence for therapeutic studies from the Centre for Evidence-Based Medicine, Oxford [7] (Table 1). Disagreements were resolved through discussion.

**Table 1:** Quality assessment for the included studies using the Oxford Centre for Evidence-based Medicine – Levels of Evidence criteria [7].

Author(s)	Study Type	Level of Evidence
Jauhari et al., 2015	RCT	2b
Kaushik et al., 2016	RCT	2b
Nagilla et al., 2017	RCT	2b
Sezgin et al., 2019	RCT	2b

## 3 Results

### 3.1 List of excluded studies

Figure 1 shows the search strategy that was employed to gather relevant publications for this review. Following the initial search, we considered thirty-eight studies to be potentially eligible for inclusion, but after comprehensive screening of the full articles, thirty-four were excluded for not meeting the inclusion criteria for this review (Table 2). The four papers were then subsequently analysed for data extraction.

**Table 2:** Full text articles excluded and reason for exclusion.

Author(s)	Year	Reason for Exclusion
Vadhana et al. [18]	2019	Incorrect intervention
Sheikh and Iyer [19]	2016	Incorrect intervention
Gbinigie et al. [20]	2016	Review
Puri [21]	2015	Opinion paper
Telles et al. [22]	2009	Letter
Penmetsa and Pitta [23]	2019	Incorrect intervention
Kandaswamy et al. [24]	2018	Incorrect intervention
King [25]	2018	Review
Naseem et al. [26]	2017	Review
Shanbhag et al. [2]	2017	Review
Howshigan et al. [27]	2015	Incorrect intervention
Kuroyama et al. [17]	2015	Incorrect intervention
Sood et al. [28]	2014	Incorrect intervention
Oklahoma Dental Association [29]	2014	Letter
Singh et al. [13]	2011	Review
Asokan et al. [30]	2011	In vitro study
Asokan et al. [31]	2008	Incorrect intervention
Karthikeson [32]	2019	Survey
Jeevan et al. [33]	2019	Review
Swathi and Maragathavalli [34]	2018	Review
Seher et al. [35]	2017	Incorrect intervention
Mathewand Sankari [36]	2014	Review
Lakshmi et al. [37]	2013	Review
Mittal et al. [38]	2018	Incorrect intervention
Asokan et al. [30]	2011	Incorrect intervention
Asokan et al. [39]	2009	Incorrect intervention
Wong et al. [15]	2018	Incorrect intervention
Asokan [40]	2008	Letter
Shetty [41]	2019	Unable to access journal
Kablian and Ramamurthy [42]	2016	Incorrect intervention
Halim et al. [43]	2014	Full text unavailable
Shino et al. [44]	2015	In vitro study
Lavine et al. [45]	2018	In vitro study
Dewi et al. [46]	2017	In vitro study
Shanbhag [2]	2017	In vitro study
Peedikayil et al. [47]	2016	Non-RCT
Zope [48]	2017	Non-RCT
Peedikayil et al. [3]	2015	Non-RCT

### 3.2 Analysis of measured outcomes

A total of four randomised-controlled studies were included in this systematic review [8, 9, 10, 11] (Tables 3 and 4). All the published data described patients treated between 2015 and 2019. The total number of subjects involved in these four studies was 182. The age range of the participants was between 6 and 52 years. Only three studies reported a mean age [8, 9, 10]. The mean for this review was 22.3 years.

**Table 3:** Study characteristics from studies included in systematic review.

Author(s)	Design	*n*	Age range	Mean age	Study duration	Intervention	Oral hygiene adjustment	Control	Outcomes Measured	Funding
Jauhari et al., 2015	RCT	52	6–12 years	NR	14 days	Coconut oil	Oil pulling twice daily	Distilled water. Mouthrinse twice daily	1. Oral microbial levels 2. *S. Mutans* level in saliva	None
Kaushik et al., 2016	RCT	60	18–22 years	20	14 days	Coconut oil	Oil pulling 10ml for 10 min	Distilled water. Mouthrinse 5ml for 1 min	1. Microorganism total colony-forming units	None
Nagilla et al., 2017	RCT	40	18–22 years	20.5	7 days	Coconut oil	Oil pulling 10–15ml for 10 min. No toothbrushing	Mineral water. Mouthrinse. No toothbrushing	1. Plaque index	None
Sezgin et al., 2019	RCT	30	18–52 years	26.3	14 days	Coconut oil	Oil pulling 10ml twice daily for 15–20 min	Chlorhexidine 0.2%. Mouthrinse 10ml twice daily for 30 s	1. Plaque index 2. Stain index 3. Gingival index 4. Bleeding on probing	Baskent University Research Fund, Turkey

**Table 4:** Reported outcomes from studies included in systematic review.

Author(s)	*n*	PI	GI	BOP	SI	Salivary *SM* count	Salivary BC count
Jauhari et al.	52	NR	NR	NR	NR	Change in mean score OIL: 0.54 (0.967); *p* = 0.068 CTRL: 0.41 (0.796) *p* = 0.078 Comparison of change in between groups *p* = 0.743	Change in mean score OIL: 10 (4.34); *p* = 0.097 CTRL: -2.31 (1.15) *p* = 0.291
Kaushik et al.	60	NR	NR	NR	NR	NR	Change in mean score OIL: 29.70 (54.82); *p* = **0.0256** CTRL: 0.90 (1.17) *p* = 0.0027 Comparison of change in between groups ***p* = 0.05**
Nagilla et al.	40	Post intervention score OIL: 1.16 (0.28) CTRL: 1.50 (0.37) ***p*=<0.001**	NR	NR	NR	NR	NR
Sezgin et al.	30	Post intervention score OIL: 1.67 (0.24) CHX: 1.61 (0.20) *p* = 0.09	Post intervention scoreOIL: 0.60 (0.21) CHX: 0.67 (0.25) *p* = 0.286	Post intervention score OIL: 0.09 (0.30) CHX: 0.01 (0.09) *p* = 0.225	Post intervention scoreOIL: 0.21 (0.13) CHX: 0.47 (0.27) ***p* = 0.0002**	NR	NR

Abbreviations: n (number of participants); PI (plaque index); GI (gingival index); SI (stain index); SM (Streptococcus mutans); BC (bacterial colony); NR (not reported); OIL (coconut oil pulling group); CTRL (control group); CHX (chlorhexidine digluconate).

Bold: Statistically significant (≤ 0.05).

All four studies used coconut oil as an intervention for oil pulling (OIL). Three studies used distilled or mineral water as a control group (CTRL) [9, 10, 11] and one study compared the use of chlorhexidine digluconate (CHX) 0.2% with the coconut oil pulling intervention [8]. The oral hygiene adjustment differed for each study. Two advised oil pulling for 10 min [9,10], however one of these advised no toothbrushing [9]. One study advised oil pulling for 15–20 min [10] whereas the other study had no time limit set but advised oil pulling to be carried out twice daily [11]. Apart from one study conducted over 7 days [9], the duration of the remaining studies were all 14 days [8,10,11].

#### 3.2.1 Plaque index score

Two studies reported data on the plaque index score [8,9]. Nagilla et al. found a statistically significant difference between the control group (CTRL) and coconut oil pulling intervention (OIL) (*p*=<0.001). Sezgin et al. found no significant difference in the reduction of plaque index score between the OIL group and chlorhexidine group (CHX) after 14 days (*p* = 0.09).

#### 3.2.2 Gingival index score

One study assessed gingival index score [8]. Sezgin et al. found no significant difference in the gingival index score between the OIL group and CHX after 14 days (*p* = 0.286).

#### 3.2.3 Bleeding on probing

One study assessed bleeding on probing [8]. Sezgin et al. found no significant difference in the gingival index score between the OIL group and CHX after 14 days (*p* = 0.225).

#### 3.2.4 Stain index

One study assessed stain index [8]. Sezgin et al. found the CHX group exhibited higher scores (increased tooth staining) compared to OIL and the differences between the two groups were statistically significant (*p* = 0.0002).

#### 3.2.5 Salivary Streptococcus mutans *count*

One study assessed salivary Streptococcus mutans count [11]. Jauhari et al. found there was no statistical difference for both the OIL group (*p* = 0.967) and control group (*p* = 0.796) with regards to the change in Streptococcus mutans count after 14 days. In addition, there was no statistically significant difference between these two groups (p = 0.743).

#### 3.2.6 Salivary bacterial colony count

Two studies reported the total salivary bacterial colony counts [10, 11]. Jauhari et al. found there was reductions in the bacterial colony count for the OIL group, however there was no statistically significant difference with this result (*p* = 0.097). No comparison was reported with the control group. In comparison, Kaushik et al. found with regards to the reduction in the total bacterial colony count, there was a statistically significant difference for the OIL group (*p* = 0.0256). In addition, there was a statistically significant difference between the reduction in total salivary bacterial colony count between the OIL and control groups (*p* = 0.05).

### 3.3 Risk of bias and review quality assessment

There was a significant variation in the presence of bias within all four studies (Figures 2 and 3). Due to the nature of the intervention and coconut oil having a distinct taste and consistency, it was expected that a number of the studies would have a high-level of risk of performance bias. Only one study demonstrated that measures had been sufficiently undertaken to adequately reduce this level of risk regarding the blinding of participants [9]. Selection bias was another area of concern. It was unclear in three studies whether the allocation of groups had been concealed.

The quality of the studies was assessed using the Oxford Levels of Evidence (Figure 3). All RCTs were deemed to be of low quality due to the to the lack of statistical analysis of the results including no odds ratios or confidence intervals. For this reason, one cannot be confident that the results of the interventions are near the true value for the outcomes, across all four studies. All studies reported no conflict of interest and all, bar one [8], had no source of funding (Figure 1).

## 4 Discussion

In Ayurvedic medicine, oil pulling is claimed to cure more than thirty systemic diseases ranging from diabetes to asthma [12]. It has been used extensively for many decades in the Indian subcontinent and now has a global presence. Oil pulling therapy is traditionally carried out using sesame oil, but other oils such as sunflower and coconut oil have been advocated [13]. Other systematic reviews have considered the effect of sesame and alternative oils on dental hygiene and oral health, however to the best of our knowledge, this is the first systematic review to assess the effect of coconut oil for oil pulling on oral health.

The results from the included randomised controlled trials demonstrated evidence that coconut oil pulling has a significant effect on plaque index score when compared to the control group. The evidence for coconut oil pulling having a reduction in salivary bacterial colony count was variable. Both studies detected a reduction, however there was no reported statistical difference in one. With regards to salivary Streptococcus mutans count, the evidence suggests that coconut oil pulling has no change when compared to a control after two weeks of the intervention.

One study compared the use of chlorhexidine mouthwash, a broad-spectrum antiseptic used frequently in the management of gingivitis and periodontitis [14]. The evidence suggests that chlorhexidine mouthwash use has no statistical difference compared to the use of coconut oil pulling for plaque score; gingival index score and bleeding-on-probing. Predictably, there was a significant difference in staining when comparing these two groups. As a well understood side effect of chlorhexidine, hard-tissue staining poses an issue for both patients and for dental care professionals with regards to removal. Chlorhexidine mouthwash has been reported to have a number of other adverse effects, most commonly taste disturbance; hypersensitivities and mucosal soreness or irritation [14]. Supporters of coconut oil pulling may see these adverse effects of using chlorhexidine mouthwash as another reason to promote the use of coconut oil; unfortunately none of these effects were demonstrated in the included studies, most likely due to the short study durations.

No studies included in this review reported on the adverse effects of oil pulling. Throughout the full-text screening, a small number of articles described cases of lipid pneumonia in patients who regularly oil pulled [15, 16, 17]. However, in all case reports, the patients were reported as suffering with swallowing dysfunctions and or were at a high risk of aspiration. Nevertheless, as there has been no definitive evidence published on this adverse effect, careful consideration is needed.

The results from this review must be interpreted with caution. Evidence has been concluded from a small sample of RCTs that are not well powered. The small sample sizes and short durations of interventions could have affected the sensitivity of the results and thus drawn misleading conclusions.

Furthermore, inter-study variability in methodology made it for difficult for grand comparisons to be made. Coconut oil was used as the method of intervention for all studies. In three studies, this was compared to water as a control, and the other study used chlorhexidine. Only one study detailed clearly the complete oral hygiene adjustment during the study period and documented the advice given to participants to stop brushing for the study duration [9]. It was unclear what other oral hygiene habits were enforced in the other three studies.

A robust search strategy was carried out adhering to PRISMA guidelines however we recognise a number of limitations of this systematic review. Firstly, due to the small sample size and short duration of the studies reported, it is unclear whether these results can be extrapolated and applied to long-term effects. In addition, three of the studies were conducted in the same country; India, and all were conducted in the Asian continent and it is therefore not appropriate to apply these findings other regions. In addition, due to the nature of the differences in inter-study methodology, quantitative pooling of results was unachievable and therefore distinct correlations and corresponding conclusions cannot be made.

Secondly, despite the measures in place to avoid bias within these studies, owing to the very nature of the interventions with variable coconut oil having a distinct taste, colour and consistency, complete subject blindness is difficult and therefore with both selection and performance biases, results may have been misleading. This was evident from the assessment of the risk of bias (Table 1, Figure 2). Finally, despite a comprehensive search strategy, it may be the case that other randomised controlled trials exist that have not been published.

The authors believe that additional randomised controlled trials are necessary to determine whether coconut oil pulling improves oral health and if so; the mechanism of action. The authors advocate, in general, that the following rules should be applied for future studies:

• Studies should be conducted in multiple centres with a larger sample population.
• Outcomes should be assessed with standardised reproducible scales and should be calibrated amongst the clinicians involved in the study.
• Studies should be carried out and described in sufficient detail to allow an assessment of comparable groups.
• Common, quantifiable and clinically relevant data (time of intervention, oral hygiene adjustments, specific outcomes, treatment acceptability and participant satisfaction) should also be included in a sufficiently detailed manner.
• A longer study duration should be used.
• A follow-up period is essential to identify a predictable treatment effect.

## 5 Conclusion

This is the first systematic review reporting the effect of coconut oil when used for oil pulling to improve dental hygiene and oral health. This study has observed and highlighted the absence of high-quality evidence in the literature subjected to bias. Consequently, it is therefore difficult to determine whether oil pulling with coconut oil has an actual beneficial effect. It is promising to see beneficial outcomes and the authors hope this review will encourage further research to a higher quality in the future. To conclude, the available data suggests that a larger number of well-designed randomised controlled trials are essential to determine the impact of oil pulling with coconut oil on oral health.

## Declarations

### Author contribution statement

J. Woolley and T. Gibbons: Conceived and designed the experiments; Performed the experiments; Analyzed and interpreted the data; Wrote the paper.

K. Patel and R. Sacco: Analyzed and interpreted the data; Wrote the paper.

### Funding statement

This research did not receive any specific grant from funding agencies in the public, commercial, or not-for-profit sectors.

### Competing interest statement

The authors declare no conflict of interest.

### Additional information

No additional information is available for this paper.
